# Multigene phylogeny and taxonomic revision of American shrimps of the genus *Cryphiops* Dana, 1852 (Decapoda, Palaemonidae) implies a proposal for reversal of precedence with *Macrobrachium* Spence Bate, 1868

**DOI:** 10.3897/zookeys.1047.66933

**Published:** 2021-07-01

**Authors:** Fernando L. Mantelatto, Leonardo G. Pileggi, João A. F. Pantaleão, Célio Magalhães, José Luis Villalobos, Fernando Álvarez

**Affiliations:** 1 Laboratório de Bioecologia e Sistemática de Crustáceos (LBSC), Departamento de Biologia, Faculdade de Filosofia, Ciências e Letras de Ribeirão Preto (FFCLRP), Universidade de São Paulo (USP), Ribeirão Preto, São Paulo, Brazil Universidade de São Paulo São Paulo Brazil; 2 Instituto Nacional de Pesquisas da Amazônia (INPA) (Retired), Manaus, Amazonas, Brazil Instituto Nacional de Pesquisas da Amazônia Amazonas Brazil; 3 Colección Nacional de Crustáceos, Instituto de Biología, Universidad Nacional Autónoma de México, Apartado Postal 70-153, 04510 Ciudad de México, Mexico Universidad Nacional Autónoma de México Mexico Mexico

**Keywords:** Freshwater decapods, genetic variability, *
Macrobrachium
*, molecular phylogeny, Neotropical region, prawn

## Abstract

The freshwater shrimp genus *Cryphiops* Dana, 1852 has a disjunct distribution in North (Mexico) and South (Brazil, Chile) America, and is composed of only six species. The current classification of genera in the Palaemonidae is controversial, based on variable morphological characters, and still far from a clear definition. *Cryphiops* differs from the speciose genus *Macrobrachium* Spence Bate, 1868 only by the absence of the hepatic spines on the carapace. Previous studies with a limited dataset suggested the necessity to link morphology and phylogeny to create an internal rearrangement in the genus to resolve the paraphyletic status. Through a molecular phylogenetic approach, the evolutionary relationships are inferred based on four (mitochondrial and nuclear) genes, among all recognized species of *Cryphiops* and, in combination with a taxonomic revision, a rearrangement in the systematics of the genus is suggested. The absence of hepatic spines on the carapace, the only character used to separate the genus *Cryphiops*, is subjective and should be considered as a homoplasy. This implies that *Cryphiops* and *Macrobrachium* are subjective synonyms and, because the latter genus is much more diverse and widely known, with several economically important species, to avoid confusion and disturbance in nomenclatural stability and keep universality, a proposal for the priority of the older synonym (*Cryphiops*) to be partially suppressed in favor of maintaining the prevailing use of the younger synonym (*Macrobrachium*) is presented. As the species of *Cryphiops* should be accommodated in the genus *Macrobrachium*, new names to replace three preoccupied specific names that, by this action, resulted to be secondary homonyms are offered.

## Introduction

During the 2010s, caridean shrimp systematics has undergone considerable changes at different levels (see De Grave et al. 2015a for review of the context and literature). The speciose Palaemonidae Rafinesque, 1815 is an example of this new tendency. The family consists of a large group of decapod crustaceans comprising 151 genera and approximately 780 species (WoRMS 2021), which reached a great evolutionary success, occupying marine, estuarine, and freshwater environments. Members of this group have a long taxonomic history and it can be considered a challenge to build a more natural classification since their morphology is highly conservative (Holthuis 1950, 1952a; Pereira 1997; Murphy and Austin 2005; Pileggi and Mantelatto 2010; De Grave and Ashelby 2013). Recently, considerable efforts have been taken to solve taxonomic incongruences and accommodate taxa in a more consistent classification (De Grave et al. 2009; De Grave and Fransen 2011). After the construction of this major guideline, some important specific and complementary taxonomic initiatives were developed focusing on different taxa (for a review see De Grave et al. 2015a). Despite this significant advance, the current knowledge is not sufficient to cover the tremendous diversity of palaemonids and the many questions that remain unanswered. One of these unsolved problems is that of *Cryphiops* Dana, 1852, a genus composed by six recognized species distributed in North (Mexico) and South (Brazil and Chile) America (Villalobos Hiriart et al. 1989; Baldari et al. 2010). Of the six species, only Cryphiops (C.) caementarius (Molina, 1782) needs estuarine water to complete its reproductive cycle while the other five [Cryphiops (Bithynops) brasiliensis Gomes Corrêa, 1973, Cryphiops (Bithynops) luscus (Holthuis, 1973), Cryphiops (Bithynops) perspicax (Holthuis, 1977), Cryphiops (Bithynops) sbordonii Baldari, Mejía-Ortiz & López-Mejía, 2010, and Cryphiops (Bithynops) villalobosi Villalobos Hiriart, Nates Rodríguez & Cantú Díaz Barriga, 1989] are restricted to inland waters with no apparent dependency of estuarine environments.

The taxonomic reappraisal of *Cryphiops* showed a close relationship with *Macrobrachium* Spence Bate, 1868, from which *Cryphiops* only differs by the absence of the hepatic spines on the carapace (Holthuis 1950, 1952a). The absence of one or both spines was also encountered by Short (2004) in some Australian species of *Macrobrachium* [*M. hendersoni* (De Man, 1906), *M. hildebrandti* (Hilgendorf, 1893), *M. pilimanus* (De Man, 1879), and *M. koombooloomba* Short, 2004] who offered no explanation about the evolutionary importance of this character. Thus, considering the subjectivity of the character separating *Cryphiops* and *Macrobrachium*, it is imperative to conduct further in-depth studies, using different approaches, to resolve their relationship.

Molecular phylogenetic analysis including species of *Cryphiops* are scarce and appear only as part of broader studies with different objectives, for example Porter et al. (2005) and Page et al. (2008). Both studies presented *C. caementarius* nested within *Macrobrachium*. Further, in a molecular phylogenetic study of *Macrobrachium*, Pileggi and Mantelatto (2010) recovered the genus as a monophyletic clade if representatives of *Cryphiops* (*C. brasiliensis* and *C. caementarius*) were included. In addition, these authors pointed out two important aspects: first, the phylogenetic positioning regarding the type of larval development presented by both species of *Cryphiops* and, second, that the character used to separate both genera, the presence of the hepatic spine, is indeed subjective and should be reconsidered in a future revision.

Pereira (1997), using a cladistic analysis based on morphologic characters, stated that phylogenetic studies would be necessary to promote an internal rearrangement of the subgroups of Palaemonidae, because many of these proved to be paraphyletic (e.g., *Palaemonetes*, *Palaemon*, *Macrobrachium*, *Cryphiops*, *Bithynops*). Only recently some of these genera have been studied and undergone taxonomic changes, as was the case of *Palaemonetes* Heller, 1869 and *Palaemon* Weber, 1795 (see De Grave and Ashelby 2013 and Carvalho et al. 2017, 2020 for literature and details).

Thus, considering that the taxonomic status of this group is not yet fully resolved, and that no systematic rearrangement has been proposed, we used a multigene phylogenetic approach to assess the relationships among all species of *Cryphiops* in comparison with species of *Macrobrachium* from America, Africa, and the Indo-Pacific and, along with a taxonomic revision, we propose a rearrangement in the systematics of the group.

## Materials and methods

### Sample collection

Fresh specimens (*Cryphiops* and additional taxa) for molecular analysis were obtained from field collections in rivers and estuaries in Brazil, Chile, Venezuela, Costa Rica, and Mexico (Table 1). The new individuals were preserved in 75–90% ethanol. Additional material was obtained through donations, visits, or loans from various crustacean collections around the world (Table 1).

### Repositories

Material examined is deposited in the Crustacean Collection of the Department of Biology (**CCDB**), Faculty of Philosophy, Sciences and Letters at Ribeirão Preto (**FFCLRP**), University of São Paulo (**USP**), Brazil; National Crustacean Collection (**CNCR**), of the Institute of Biology, Universidad Nacional Autónoma de Mexico (**UNAM**), Mexico; and Museu Nacional, Universidade Federal do Rio de Janeiro (**MNRJ**), Brazil.

### Molecular data

The molecular analysis was based on partial fragments of the 16S rDNA, COI mtDNA, 18S nDNA, and H3 nDNA genes, which have been effective in solving different levels of relationships among decapod species (Schubart et al. 2000; Porter et al. 2005; Pileggi and Mantelatto 2010; Mantelatto et al. 2011; Vergamini et al. 2011; Carvalho et al. 2013, 2017; Rossi and Mantelatto 2013; Álvarez et al. 2020; Robles et al. 2020).

DNA extraction, amplification and sequencing protocols followed Pileggi and Mantelatto (2010). Total genomic DNA was extracted from muscle tissue of the walking legs, chelipeds, or the abdomen. The amplification by polymerase chain reaction (PCR) was conducted with the following primers: 16Sar and 16Sbr (Palumbi et al. 1991) for the 16S mitochondrial gene; COI-a and COI-f (Palumbi and Benzie 1991) for the COI mitochondrial gene; 18Sai and 18Sb3.0 (Whiting et al. 1997) for the 18S nuclear gene; H3ar and H3af (Colgan et al. 1998) for the histone (H3) nuclear gene. PCR products were sequenced with the ABI Big Dye Terminator Mix (Applied Biosystems, Carlsbad, CA) in an ABI Prism 3100 Genetic Analyzer (Applied Biosystems automated sequencer) following Applied Biosystems protocols. All sequences were confirmed by sequencing both strands. Genetic vouchers generated were deposited in the CCDB and CNCR under the catalogue numbers listed in Table 1.

**Table 1.** *Cryphiops* and *Macrobrachium* species used for molecular techniques. CCDB: Collection of Crustaceans, Department of Biology, Faculty of Philosophy, Sciences and Letters of Ribeirão Preto, University of São Paulo, Brazil; CIB: Crustacean collection at Centro de Investigaciones Biológicas del Noroeste (CIBNOR), Mexico; CNCR: National Crustacean Collection, UNAM, México; GU: Griffith University, Australia; JC: Monte L. Bean Life Science Museum, Brigham Young University, Provo, Utah. USA; INPA: Instituto de Pesquisa da Amazônia, Brazil; MPEG: Museu Paraense Emilio Goeldi, Brazil; MZUCR: Museum of Zoology, University of Costa Rica, Costa Rica; UFRGS: Collection of Crustaceans, Federal University of Rio Grande do Sul, Brazil; OUMNHC-ZC: Zoological Collections, Oxford Museum of Natural History, UK; RMNH: Naturalis Biodiversity Center (former Rijksmuseum van Natuurlijke Historie), The Netherlands. Other abbreviations: AM, state of Amazonas; AP, state of Amapá; DF, Distrito Federal; PA, state of Pará; PR, state of Paraná; SP, state of São Paulo; USA, United States of America.

Species	Locality	Collection code and catalogue #	GenBank #			
			(16S)	(COI)	(18S)	(H3)
*Cryphiops* new status						
***M. alevillalobosi* nom. nov., comb. nov.**	Ocosingo, Chiapas, Mexico	CNCR 3650b	–	–	MZ413044	–
	Ocosingo, Chiapas, Mexico	CNCR 5760	JF491348	–	–	–
***M. caementarius* (Molina, 1782) comb. nov.**	Coquimbo, Chile	CCDB 1870	HM352453	HM352495	KM101490	–
	Chile	JC 1219	DQ079711	–	DQ079747	DQ079672
***M. candango* nom. nov., comb. nov.**	Brasília-DF, Brazil	CCDB 2195	HM352434	–	–	–
	Brasília-DF, Brazil	CCDB 5894	MZ413047	–	MZ413038	MZ403772
	Brasília-DF, Brazil	CCDB 5897	MZ413048	–	MZ413039	MZ403773
***M. luscus* (Holthuis, 1973) comb. nov.**	La Trinitaria, Chiapas, Mexico	CNCR 5759	JF491343	MZ423177	MZ413040	–
***M. perspicax* (Holthuis, 1977) comb. nov.**	La Trinitaria, Chiapas, Mexico	CNCR 7898	MZ413049	MZ423178	MZ413041	–
	La Trinitaria, Mexico	CNCR 25392	MZ413050	MZ423179	MZ413042	MZ403775
***M. valdonii* nom. nov., comb. nov.**	La Trinitaria, Chiapas, Mexico	CNCR 25108	–	MZ423180	MZ413043	MZ403776
Comparative species						
*M. acanthurus*	Guaraqueçaba-PR, Brazil	CCDB 2546	HM352444	KM101538	KM101493	–
	Bocas del Toro, Panama	CCDB 3538	KM101467	KM101541	KM101496	–
*M. amazonicum*	Santana-AP, Brazil	CCDB 1965	HM352441	HM352486	KM101497	–
	Panama	CNCR 5151	KM101468	KM101542	KM101498	–
*M. americanum*	Puntarenas, Costa Rica	CCDB 2883	JQ805797	JQ805899	JQ805843	JQ805861
	Puntarenas, Costa Rica	MZUCR 3292-03	KM101473	KM101547	KM101504	–
	Isla Violines, Costa Rica	MZUCR 2970-01	KM101472	KM101546	KM101503	–
*M. australe*	Hualien, Taiwan	Not informed	DQ194904	AB235245	–	–
	Not informed	Not informed	–	–	GU204997	–
	Not informed	GU 363	–	–	–	FN995544
*M. borellii*	Buenos Aires, Argentina	UFRGS 3669	HM352426	HM352480	KM101505	–
*M. brasiliense*	Serra Azul-SP, Brazil	CCDB 2135	HM352429	HM352481	KM101506	–
*M. carcinus*	Santana-AP, Brazil	CCDB 2122	HM352448	HM352490	KM101507	–
	Isla Margarita, Venezuela	CCDB 2123	HM352450	HM352492	KM101508	–
	Cahuita, Costa Rica	CCDB 2145	HM352452	KM101548	KM101510	–
*M. crenulatum*	Isla Margarita, Venezuela	CCDB 2124	HM352463	HM352498	KM101512	JQ805865
	Parque Veragua, Costa Rica	CCDB 4874	KM101475	KM101550	KM101513	–
*M. digueti*	Puntarenas, Costa Rica	MZUCR 3292-01	KM101476	KM101551	KM101514	–
	Oaxaca, Mexico	CNCR 24811	JQ805808	JQ805905	JQ805849	JQ805870
	Limón, Costa Rica	CCDB 2882	JQ805806	JQ805903	JQ805847	JQ805868
*M. dux*	Warri, Nigeria	Not informed	KJ463388	KC688273	–	–
*M. equidens*	Pará, Brazil (introduced)	MPEG 0809	MZ413051	MZ423181	–	–
	Not informed	Not informed	–	–	GU205009	–
	Khatib Bongsu, Singapore	Not informed	–	–	–	FM958095
*M. faustinum*	Jamaica	RMNHD 17613	JQ805809	JQ805907	JQ805850	JQ805871
*M. ferreirai*	Manaus-AM, Brazil	CCDB 2125	HM352427	HM352483	–	–
*M. gracilirostre*	Hualien, Taiwan	Not informed	DQ194924	AB235258	–	–
	Not informed	Not informed	–	–	GU205013	–
*M. gracilirostre*	Manado, Indonesia	Not informed	–	–	–	FM958099
*M. hancocki*	Puntarenas, Costa Rica	CCDB3092	JQ805814	JQ805912	JQ805851	JQ805874
	Panama	RMNHD 8810	JQ805817	JQ805915	JQ805852	JQ805876
*M. heterochirus*	Ilha de São Sebastião-SP, Brazil	CCDB 2137	HM352454	HM352494	KM101515	–
	Cahuita, Costa Rica	CCDB 2899	KM101477	KM101552	KM101516	–
	Parque Veragua, Costa Rica	CCDB 4875	KM101478	KM101553	KM101517	–
*M. hobbsi*	Oaxaca, Mexico	CIB 1168.5	–	MH253251	–	–
	Huatabampo, Mexico	CNCR 2239	KF383306	–	–	–
*M. idae*	Khanom, Thailand	Not informed	DQ194930	AB235262	–	–
	Not informed	Not informed	–	–	GU205019	–
	Tioman, Malaysia	Not informed	–	–	–	FM958103
*M. iheringi*	Brasília-DF, Brazil	CCDB 5899	MZ413052	MZ423182	MZ413045	–
*M. inpa*	Manaus-AM, Brazil	CCDB 2127	HM352433	–	–	–
*M. jelskii*	Pereira Barreto-SP, Brazil	CCDB 2129	HM352437	HM352484	KM101519	–
*M. lar*	French Polynesian	GU 992	EF588316	–	–	EU249462
	Ryukyus, Japan	Not informed	–	AB235269	–	–
	Not informed	Not informed	–	–	KP215302	–
*M. latidactylus*	Tioman, Malaysia	Not informed	DQ194944	AB235272	–	–
	Not informed	Not informed	–	–	GU205024	–
	Tioman, Malaysia	Not informed	–	–	–	FM958109
*M. latimanus*	Cebu, Philippines	Not informed	DQ194937	AB235276	–	
	Not informed	Not informed	–	–	GU205026	–
	Ciawi Tali, Indonesia	Not informed	–	–	–	FM958110
*M. nattereri*	Lago Tupê-AM, Brazil	CCDB 2130	HM352428	–	–	–
*M. occidentale*	Oaxaca, Mexico	CNCR 24838	KM101481	KM101556	KM101521	–
	Puntarenas, Costa Rica	MZUCR 3292-02	KM101482	KM101557	KM101522	–
*M. olfersii*	Ilha de São Sebastião-SP, Brazil	CCDB 2435	HM352459	HM352496	KM101523	–
	Isla Margarita, Venezuela	CCDB 2446	HM352460	KM101559	KM101525	JQ805886
	Parque Veragua, Costa Rica	CCDB 4873	KM101483	KM101560	KM101526	–
*M. ohione*	Louisiana, USA	CCDB 4304	MZ413053	MZ423183	MZ413046	MZ403774
*M. panamense*	Guanacaste, Costa Rica	MZUCR 2971-01	KM101484	KM101561	KM101527	–
	Guanacaste, Costa Rica	MZUCR 3291-01	KM101486	KM101563	KM101529	–
*M. potiuna*	Eldorado-SP, Brazil	CCDB 2131	HM352438	KM101564	KM101530	–
	Cananéia-SP, Brazil	CCDB 3652	JX466936	–	KP179011	KP179067
*M. rosenbergii*	Jaboticabal-SP, Brazil (Culture)	CCDB 2139	HM352465	–	KM101531	–
	Kaohsiung Co., Taiwan	Not informed	–	AB235295	–	–
	Irian Jaya, Indonesia	Not informed	–	–	–	FM958123
*M. surinamicum*	Icangui-PA, Brazil	INPACR 183	HM352446	KM101565	KM101532	–
*M. tenellum*	Oaxaca, México	CNCR 24831	KM101487	KM101566	KM101533	–
	Guanacaste, Costa Rica	MZUCR 3290-01	KM101489	KM101568	KM101535	
*M. totonacum*	Oaxaca, Mexico	CNCR 19915	KF383311	–	–	–
*M. tuxtlaense*	Veracruz, Mexico	CNCR 13174	KF383312	–	–	–
*M. vollenhoveni*	Badagry, Nigeria	Not informed	KJ463387	KC688272	–	–
*Palaemon argentinus*	Mar del Plata, Argentina	CCDB 3312	KP178997	–	KP179016	KP179115
	Mar del Plata, Argentina	CCDB 2011	HM352425	–	KM101536	–
*Palaemon modestus*	Kalkan, Kazakhstan	OUMNH-ZC 2012-01-0068	KP178986	–	KP179040	KP179099
	Jiangxi, China	Not informed	–	AB235307	–	–
*Palaemon orientis*	Kisarazu, Japan	OUMNH-ZC 2011-11-0028	KP178987	–	KP179044	KP179100
	Japan	Not informed	–	AB235306	–	–

### Molecular analyses

Edition of sequences and *denovo* assembling were carried out with the computational program Geneious v2020.2.4 (Kearse et al. 2012). COI consensus sequences (protein-coding sequence) were checked for pseudogenes by translating them and checking for indels and stop codons (Song et al. 2008). We downloaded additional *Macrobrachium* and *Palaemon* (as outgroup) species sequences available from GenBank (*Cryphiops caementarius* – DQ079711, DQ079747, DQ079672; *Macrobrachium australe* Guérin-Meneville – DQ194904, AB235245, GU204997, FN995544; *M. dux* (Lenz) – KJ463388, KC688273; *M. equidens* (Dana) – GU205009, FM958095; *M. gracilirostre* (Miers) – DQ194924, AB235258, GU205013, FM958099; *M. hobbsi* (Villalobos Hiriart and Nates Rodriguez) – MH253251; *M. idae* (Heller) – DQ194930, AB235262, GU205019, FM958103; *M. lar* (Fabricius) – EF588316, AB235269, KP215302, EU249462; *M. latidactylus* (Thallwitz) – DQ194944, AB235272, GU205024, FM958109; *M. latimanus* (von Martens) – DQ194937, AB235276, GU205026, FM958110; *M. rosenbergii* (De Man) – AB235295, FM958123; *M. vollenhovenii* (Herklots) – KJ463387, KC688272; *Palaemon modestus* (Heller) – AB235307; *P. orientis* (Holthuis) – AB235306) (Table 1). Sequences were aligned using MAFFT v.7 (Katoh and Standley 2013) with default parameters, resulting in final alignments of ~540 base pairs (bp) for 16S rDNA, ~570 bp for COI mtDNA, ~550 bp for 18S nDNA and ~330 bp for H3 nDNA. The Maximum Likelihood (ML) approach was conducted in IQ-TREE (Nguyen et al. 2015) performed in the online platform Cyberinfrastructure for Phylogenetic Research (CIPRES) (Miller et al. 2010). The evolutionary model that best fitted the data (best fit model 16S: TPM3+F+I+G4; COI: TN+F+I+G4; 18S: TIM2e+I+G4; H3: TNe+I+G4) was determined by IQ-TREE, using Bayesian Information Criterion (BIC) (Luo et al. 2010). Branch support was assessed by ultrafast bootstrap with 1,000 replicates. Genetic distances were computed for each gene using Kimura-2-parameter (K2P) in MEGA-X (Kumar et al. 2018).

In total, 88 specimens were used for the analyses, eleven belonging to *Cryphiops*, 71 to *Macrobrachium* and six to *Palaemon*, to obtain a robust representation of the ingroup and a consistent rooting of the phylogeny (Table 1). The selection of species composing both internal and external groups was based on the phylogenies proposed from morphological (Pereira 1997) and molecular characters (Murphy and Austin 2005; Liu et al. 2007; Pileggi and Mantelatto 2010). With this careful selection we covered all possible close and related species to *Cryphiops* reported previously, either by morphology and/or molecular affinities.

### Taxonomic revision

The species identification was carried by us based on diagnostic morphological features in accordance with the literature (Holthuis 1952a, 1993; Gomes Corrêa 1973; Villalobos Hiriart et al. 1989; Baldari et al. 2010). We did not list all the synonyms for *Cryphiops* and *Macrobrachium* since a complete, detailed record can be found in Holthuis (1950, 1993) and De Grave and Fransen (2011). A non-exhaustive synonyms list containing post-1950 citations focused mainly on taxonomic and faunistic studies is provided for all species and it is partially based on the “Carideorum Catalogus L.B. Holthuis”, an extensive reference catalogue of scientific names of shrimps gathered by the late L.B. Holthuis during his 68 years of studying Crustacea (Fransen et al. 2010), which was digitized and kindly and unpretentiously made available by C.H.J.M. Fransen to the community of carcinologists in digital format on 7 April 2020. For pre-1952 citations regarding *C. caementarius*, see Holthuis (1952a, b).

The morphological data considered in this review for the comparative analysis of species were as follows. Measurements: total length (tl), from the anterior portion of the rostrum to the posterior portion of telson; and carapace length (cl), from the posterior margin of the orbit to the posterior margin of the carapace. Rostrum: shape, length in relation to scaphocerite, number of teeth and their distribution on the upper and lower margins. Orbit: shape of the lower margin. Scaphocerite: size and shape. Epistome: shape and arrangement. Carapace: presence of spinules, size and arrangement of hepatic and antennal spines. Pereiopods: size and shape of the first pereiopods (P1); size, shape, and proportion of the articles of the second pereiopods (P2); size and proportion of the articles of the third, fourth and fifth pereiopods (P3 to P5). Thoracic sternum: presence and shape of the median process (T4). Abdomen: surface roughness, shape of the pleura of the fifth somite. Pleopods: ratio appendix masculina/appendix interna of the second pair (PL2). Pre-anal keel: presence and shape in the inter-uropodal sclerite. Uropods: presence of external spines. Telson: general shape, shape of the posterior margin, presence, and distribution of dorsal spines, positioning of the posterior spines in relation to the posterior margin. Other aspects such as the size of males and ovigerous females, life cycle, color, distribution, systematic position, type locality and general considerations were also considered.

## Results

### Molecular approach

The concatenated phylogenetic analysis included 45 species of Palaemoninae: six belonging to *Cryphiops*, 36 to *Macrobrachium*, and three to *Palaemon*. A total of 35 new DNA sequences was generated in this study: seven 16S and seven COI mitochondrial sequences, ten 18S, and eleven H3 nuclear sequences. The final alignment of the four markers totalized 1,982 bp.

**Figure 1.**
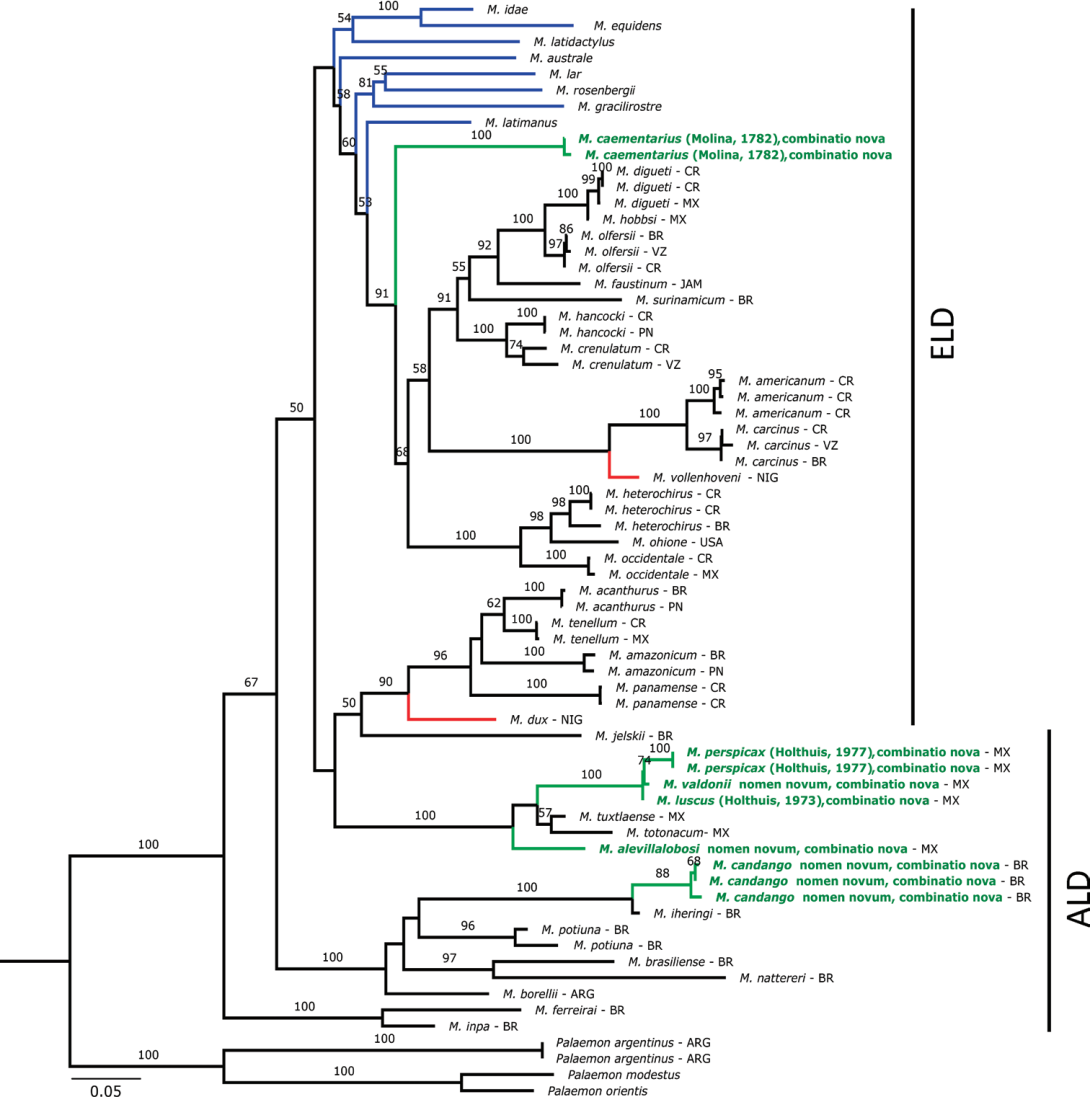
Concatenated phylogenetic tree of selected species of *Macrobrachium* representing wide geographical distribution of the group, based on the method of maximum likelihood of the 16S rDNA, COI mtDNA, 18S nDNA and H3 nDNA genes. Numbers on nodes refer to significance values of 1000 bootstrap replicates; values ≤ 50% are not shown. ARG: Argentina; BR: Brazil; CH: Chile; CR: Costa Rica; JAP: Japan; MX: México; NIG: Nigeria; PN: Panamá; VZ: Venezuela. ALD: Abbreviated larval development. ELD: Extended larval development. Blue: species from Indo-Pacific. Red: species from Africa. Green: former *Cryphiops* species.

The topology obtained by ML with the four concatenated genes (Fig. 1) showed a clear positioning of the genus *Cryphiops* nested among the species of *Macrobrachium* as was also found in previous studies (Pereira 1997; Porter et al. 2005; Page et al. 2008; Pileggi and Mantelatto 2010). Genetic distances found among species of *Macrobrachium* and *Cryphiops* corroborate this inclusion (Suppl. material 1). The levels of divergence ranged from 2.8% to 18.8% (*Cryphiops* spp., *Macrobrachium* spp.) and from 0.23% to 15.1% among *Cryphiops*, for 16S; from 18.2% to 27.3% (*Cryphiops* spp., *Macrobrachium* spp.) and from 0.73% to 26.0% among *Cryphiops*, for COI; from 0.4% to 9.0% (*Cryphiops* spp., *Macrobrachium* spp.) and from 0.2% to 4.7% among *Cryphiops*, for 18S; and from 2.2% to 12.1% (*Cryphiops* spp., *Macrobrachium* spp.) and from 7% to 10.7% among *Cryphiops*, for H3.

The estuarine *C. caementarius*, which has extended larval development (ELD), is nested among the species of *Macrobrachium* that have the same type of larval development (Fig. 1). Similarly, the species of *Cryphiops* from inland waters (*C. brasiliensis*, *C. luscus*, *C. perspicax*, *C. sbordonii*, and *C. villalobosi*) with abbreviated larval development (ALD) are positioned in clades with species of *Macrobrachium* that have ALD (Fig. 1). Interestingly, the recovered phylogeny follows the previous subdivision proposed by Villalobos Hiriart et al. (1989) into two subgenera based on morphological and life-cycle characters, i.e., *Cryphiops* as ELD and *Bithynops* as ALD groups. The species of *Palaemon* showed a stable position in an external branch.

### Systematic account

The following new taxonomic arrangement, including diagnoses of all six species, is provided.

#### Family Palaemonidae Rafinesque, 1815

##### Genus Macrobrachium

Taxon classificationAnimaliaDecapodaPalaemonidae

Spence Bate, 1868

AE8D4FB6-FE9F-54C8-8519-F6F4B6A3D63C

Cryphiops Dana, 1852: 18 [type species: Cryphiops spinulosomanus Dana, 1852] [based on Art. 23.9.3, ICZN 1999]. – Holthuis 1952a: 136. – Holthuis 1955: 51. – Maccagno and Cucchiari 1957: 206 (in list), 213. – Holthuis 1993: 105. – Jayachandran 2001: 14 (in key), 24. – Álvarez et al. 2011: 257.
Bithynis Philippi, 1860: 164 [type species: Bithynis longimana Philippi, 1860 (= Cryphiops caementarius (Molina, 1782))].
Macrobrachium Spence Bate, 1868: 363 [type species: Macrobrachium americanum Spence Bate, 1868]. – Holthuis 1952a: 10. – Holthuis 1955: 53. – Maccagno and Cucchiari 1957: 206 (in list), 214. – Hobbs Jr. et al. 1977: 50. – Rodríguez 1980: 113. – Holthuis 1993: 109. – Chace Jr, Bruce 1993: 8. – Jayachandran 2001: 14 (in key), 49. – Valencia and Campos 2007: 3. – Álvarez et al. 2011: 260. – Dueñas et al. 2012: 69. – Campos 2014: 56.
Eupalaemon Ortmann, 1891: 696, 697 [type species: Palaemon acanthurus Wiegmann, 1836].
Parapalaemon Ortmann, 1891: 696, 731 [type species: Palaemon dolichodactylus Hilgendorf, 1879].
Macroterocheir Stebbing, 1908: 39 [type species: Palaemon lepidactylus Hilgendorf, 1879].
Bithynops Holthuis, 1973: 135 [type species: Bithynops luscus Holthuis, 1973]. – Holthuis 1993: 102. – Hobbs Jr. et al. 1977: 46. – Jayachandran 2001: 14.
Cryphiops (Cryphiops) . – Villalobos Hiriart et al. 1989: 161.
Cryphiops (Bithynops) . – Villalobos Hiriart et al. 1989: 163. – Álvarez et al. 2011: 258.

###### Type species.

*Macrobrachium americanum* Spence Bate, 1868, subsequent designation by Fowler (1912).

###### Diagnosis

(modified from Holthuis 1950, 1952a). Body compressed, generally robust, sometimes slender. Rostrum well developed, compressed, toothed, size varying from shorter to longer than distal margin of scaphocerite. Carapace with anterolateral portion smooth or bearing numerous small spinules. Carapace armed with antennal spine; hepatic spines present in most species, branchiostegal groove present, distinct. Mandible with 3-segmented palp. All maxillipeds with well-developed exopods. Pleurobranchs on third maxilliped and all pereiopods. P1 slender. P2 more robust than other pereiopods, usually longer than entire body in adult males, left and right legs often equal in size and shape or markedly different in several species. P3–P5 with dactylus simple. P5 with propodus bearing numerous transverse rows of setae in distal part of posterior margin. PL1 with endopod much smaller than exopod, endopod of male without appendix interna. Pleon with pleurae smooth in most species or with small spinules. Uropods overreaching telson; exopod with distolateral spine, endopod unarmed. Telson elongate, subtriangular, narrowing posteriorly, with two pairs of dorsal spines, anterior pair placed in middle, posterior pair usually placed midway between anterior pair and posterior margin; posterior margin ending in sharp median point, flanked by two pairs of spines, outer pair usually shorter than inner one, inner pair overreaching apex of telson in most species. See detailed description in Holthuis (1950).

###### Remarks.

See Discussion.

##### Macrobrachium alevillalobosi

Taxon classificationAnimaliaDecapodaPalaemonidae

nom. nov. comb. nov.

B9771C5A-6046-55E2-A6F9-93D68259115F

Cryphiops (Bithynops) villalobosi Villalobos Hiriat, Nates Rodríguez and Cantú Díaz Barriga, 1989: 166, figs 1–5, 7b, d, 8c. – Villalobos-Hiriart et al. 1993: 281, table 5 (list). – De Grave and Fransen 2011: 316 (catalog). – Álvarez et al. 2011: 258, fig. 2a. – Quiroz-Martínez et al. 2014: table S1 (list). – Alvarez and Villalobos 2016: 250, table 8.1 (list).
Cryphiops villalobosi . – Baldari et al. 2010: 48, fig. 1 (map), 52, table 1. – Botello and Álvarez 2013: 776, table 1 (list). – Mantelatto et al. 2020: 916 (key).

###### Material examined.

***Holotype***: Mexico – **Chiapas** • male, tl 51.8 mm; Municipality of Ocosingo, km 140 carretera Palenque-Ocosingo, ca. 5 km NW of the town of Ocosingo, Arroyo La Laja, 24 Jan. 1985, J.L. Villalobos, J.C. Nates, A. Cantú leg.; CNCR 3650. ***Paratypes***: 1 female, tl 42.7 mm, allotype; same data as for holotype; CNCR 3650 • 16 males, tl 44.3–57.0 mm, 7 females, 26.2 to 46.0 mm, 2 ovigerous females, 38.1, 42.7 mm; same date as for holotype; CNCR 3650a.

###### Additional material examined.

Mexico – **Chiapas** • 9 males, tl 25.0–45.8 mm, 30 females, 36.8–39.2 mm; Municipality of Ocosingo, km 140 carretera Palenque-Ocosingo, ca. 5 km NW of the town of Ocosingo, Arroyo La Laja; 07 Aug 1983; J.L. Villalobos, J.C. Nates, A. Cantú leg.; CNCR 2940 • 16 males, tl 24.3–54.5 mm, 23 females, tl 17.1–27.4 mm; Municipality of Yajalón, carretera Palenque-Ocosingo, Arroyo Yajalón; 07 Aug. 1983; J.L. Villalobos, J.C. Nates, A. Cantú; CNCR 2941.

###### Description.

***Rostrum*.** Short, straight, reaching slightly beyond first third of third article of antennular peduncle; upper margin with 6–9 regularly spaced teeth, first one behind posterior edge of orbit; lower margin with 1–3 teeth.

***Cephalon*.** Scaphocerite nearly 2.6 × as long as wide, outer margin straight.

***Carapace*.** Smooth; antennal spine small, slightly overreaching lower portion of orbit; hepatic spine absent. Lower orbital angle obtuse, moderately pronounced.

***Pereiopods*.**P1: slender, reaching with distal third of carpus beyond scaphocerite; carpus slightly longer than merus; chelae 0.68 length of carpus. P2: moderately robust, with small spines, equal in form and size, reaching with distal third of merus beyond scaphocerite; ischium 0.75 length of merus; merus as long as carpus; carpus as long as palm, with slight basal constriction; propodus 2.5 × as long as dactylus, and 1.6 × as long as carpus; palm compressed, nearly 5 × as long as high; fingers 0.62 length of palm, with numerous spinules, not gapping, tips crossing, cutting edge with 3–6 teeth on proximal third in both fingers. P3–P5 with all joints covered with rows of small spinules. P3 reaching with entire dactylus beyond scaphocerite, propodus 2.5 × as long as dactylus, propodus nearly 2 × as long as carpus, propodus slightly longer than merus. P4 reaching with entire dactylus beyond scaphocerite, propodus 3 × as long as dactylus, nearly 2 × as long as carpus, propodus slightly longer than merus. P5 reaching with half-length of dactylus end of scaphocerite, propodus 3 × as long as dactylus, propodus nearly 2 × as long as carpus, propodus slightly longer than merus.

***Pleon*.** Smooth; somite 5 with posteroventral angle of pleuron acute; abdominal somite merely 2 × as long as somite 5. Inter-uropodal sclerite without keel-shaped pre-anal carinae.

***Pleopods*.**PL2 with appendix masculina less than 2 × length of appendix interna.

***Uropods*.** Exopodite with mobile spines as long as spiniform projection of outer margin.

***Telson*.** Broad, smooth, slightly longer than abdominal somite 6, bearing two pairs of dorsal spinules close to posterior margin. Posterior margin ending in moderately acute triangular point; two pairs of posterior spinules with several plumose setae, inner pair overreaching distal margin of telson.

###### Etymology.

Villalobos Hiriart et al. (1989) dedicated this species to Dr. Alejandro Villalobos Figueroa, eminent Mexican carcinologist and founder of the CNCR. We maintain this homage by just adding the first part of his name to the specific epithet.

###### Size.

See in material examined.

###### Color.

Body translucid with orange punctuations.

###### Type locality.

México, Chiapas, Municipality of Ocosingo, Arroyo La Laja, km 140 carretera Palenque-Ocosingo, ca. 5 km NW of the town of Ocosingo.

###### Distribution.

Mexico, Chiapas, in the Valle de Ocosingo, Río La Virgen, Arroyos La Laja, Maravilla, Pasilá, and Yajalón (Villalobos Hiriart et al. 1989).

###### Life cycle.

Exclusive of inland waters, therefore independent of brackish waters to complete its life cycle. The eggs are few and large: 1.3–2.4 mm (Villalobos Hiriart et al. 1989). Its larval development is not known but given the characteristics of the eggs, it should be abbreviated, following the same pattern of congeners inhabiting continental waters (Magalhães and Walker 1988; Pereira and García 1995).

###### Remarks.

The name *Macrobrachium villalobosi* was used by Hobbs Jr. (1973) for a new species from Mexico. Villalobos et al. (1989) used the same name for a new species of Cryphiops (Bithynops) also from Mexico. Since the synonymization of both genera makes these specific names secondary homonyms, *Macrobrachium alevillalobosi* is proposed as a replacement name for Cryphiops (Bithynops) villalobosi Villalobos Hiriat, Nates Rodríguez & Cantú Díaz Barriga, 1989.

*Macrobrachium alevillalobosi* nom. nov., comb. nov. differs from *M. candango* nom. nov. and *M. perspicax* comb. nov. mainly in the form, size, and proportion of the articles of the second pereiopod (Table 2). The chelipeds are long and similar in size and shape, overreaching the scaphocerite with distal third of the merus; the ischium is shorter than merus; the palm is long and cylindrical, almost five times as long as high, and the dactylus is 0.62 times the length of the palm. Finally, *M. alevillalobosi* nom. nov., comb. nov. is the only species of this group in which the appendix masculina is almost as long as the endopod of the second pleopod.

**Table 2.** Morphological comparison of key characters for the species previously included in the genus *Cryphiops* Dana, 1852.

	*M. alevillalobosi* nom. nov., comb. nov.	*M. caementarius* (Molina, 1782), comb. nov.	*M. candango* nom. nov., comb. nov.	*M. luscus* (Holthuis, 1973), comb. nov.	*M. perspicax* (Holthuis, 1977), comb. nov.	*M. valdonii* nom. nov., comb. nov.
**Rostrum**	Reaching slightly beyond first third of ultimate article of antennular peduncle, and at level of distal fourth of scaphocerite. Upper margin with 6–9 teeth regularly spaced, first one behind of posterior edge of orbit; lower margin with 1–3 teeth	Reaching or slightly beyond the first article of antennular peduncle, and at level of proximal third of scaphocerite. Upper margin with 6–8 teeth regularly spaced, first one or two behind of posterior edge of orbit; lower margin with 0–4 teeth	Reaching end of antennular peduncle, and little before the distal margin of scaphocerite. Upper margin convex over orbit, with 7 teeth, first and sometimes the second, slightly behind posterior edge of orbit; lower margin with 1 tooth	Reaching or slightly overreaching joint between second and third article of antennular peduncle, and at level of distal third of scaphocerite. Upper margin convex over orbit, with 5–8 teeth regularly spaced, first over or slightly behind posterior edge of orbit; lower margin with 0–1 tooth	Reaching joint between second and third articles of antennular peduncle, and at level of distal third of scaphocerite. Upper margin with 5–8 teeth regularly spaced, first one at level or slithy behind posterior edge of orbit; lower margin with 1 or 2, rarely 3 teeth	Almost reaching the third article of antennular peduncle, and before the distal border of scaphocerite. Upper margin with 8 teeth, lack teeth in postorbital position and on ventral margin
**Eyes**	Cornea normal and larger than the peduncle	Cornea normal and larger than the peduncle	Cornea normal and larger than the peduncle	Cornea reduced, smaller than the peduncle	Cornea normal and larger than the peduncle	Cornea with a small apical black point, smaller than the peduncle
**Scaphocerite**	2.6 × as long as wide	2 × as long as wide	2.5 × as long as wide	2.5 × as long as wide (Holthuis, 1973)	2.6 × as long as wide	2.4 × as long as wide
**Lower orbital angle**	Rounded, moderately pronounced	Rounded, pronounced, as long as antennal spine	Subacute, strongly pronounced	Obtuse, moderately pronounced	Subacute, moderately pronounced	Subacute, moderately pronounced
**Antennal spine**	In the middle of the lower orbital angle	Little below the lower orbital angle	Little below the lower orbital angle	Little below the lower orbital angle	Little below the lower orbital angle	Below the lower orbital angle
**First male pereiopod**	Reaching with distal third of carpus beyond scaphocerite	Reaching with the larger part of the carpus beyond scaphocerite	Reaching with almost half length of carpus beyond scaphocerite	Reaching with nearly entire chelae beyond scaphocerite	Reaching with nearly entire chelae or small part of carpus beyond scaphocerite	Reaching with the palm beyond scaphocerite
**Second male pereiopod**	Equal in form and size, reaching with distal third of merus beyond scaphocerite; ischium 0.75× length of merus; merus as long as carpus; carpus as long as palm, with slight basal constriction; propodus 2.5× as long as dactylus, and 1.6× as long as carpus; palm compressed, nearly 5× as long as high; fingers 0.62× length of palm	Different in form and size. Largest reaching with half of merus beyond scaphocerite; ischium, merus and carpus are covered with spinules, smaller than those of the chela; ischium more than 0.5× length of merus; merus longer than carpus; carpus slightly less 0.5× length of palm, with strong basal constriction; propodus 2.5× as long as dactylus, and 3.3× as long as carpus; palm compressed, nearly 2.3× as long as high; fingers 0.75× length of palm, little gaping	Similar in shape, different in size. Largest, reaching with distal portion of merus beyond scaphocerite; ischium nearly as long as merus; merus as long as carpus; carpus slightly shorter than palm; propodus 2.5× the length of dactylus, 2× as long as carpus; palm inflated, less than 3× as long as high; fingers 0.71× the lenght of palm	Equal in form and size, reaching with proximal third of carpus beyond scaphocerite; ischium evidently shorter than merus; merus as long as carpus; carpus as long as palm, with moderate basal constriction; propodus 2× as long as dactylus, 2× as long as carpus; palm inflated, less than 3× as long as high; fingers little longer or as long as palm	Equal in form and size, reaching with proximal third of carpus beyond scaphocerite; ischium slightly shorter than merus; merus as long as carpus; carpus as long as palm, with a moderate basal constriction; propodus 2.2× as long as dactylus, 2× as long as carpus; palm inflated, 3× as long as high; fingers slightly shorter (0.8) than palm	Subequal in size, reaching with half of carpus beyond scaphocerite; ischium 0.9× merus; carpus 0.8× as long as merus and 0.85× palm length; propodus 1.5× as long as dactylus, 2.5× as long as carpus; palm 3.3× as long as high and 0.8× of dactylus length
**Appendix masculina**	Almost as long than endopod of second pleopod. Setae thick and short	Little longer than half endopod length of second pleopod. Setae thick and short	Shorter than endopod of second pleopod.	Shorter than endopod of second pleopod. Setae thick and short	Shorter than endopod of second pleopod. Setae slender and long	Shorter than endopod of second pleopod
**Inter-uropodial sclerite**	Without keel-shaped pre-anal carinae		With strong, keel-shaped pre-anal carinae	Without keel-shaped pre-anal carinae	Without keel-shaped pre-anal carinae	Without keel-shaped pre-anal carinae

##### Macrobrachium caementarius

Taxon classificationAnimaliaDecapodaPalaemonidae

(Molina, 1782) comb. nov.

05F2E07B-7D9E-500A-87B2-5F1F4EDC92CB

Cancer caementarius Molina, 1782: 208.
Palaemon Gaudichaudii H. Milne Edwards, 1837 in H. Milne Edwards 1834–1840: 400.
Cryphiops spinuloso-manus Dana, 1852: 26.
Bithynis longimana Philippi, 1860: 164.
Macrobrachium africanum Spence Bate, 1868: 366, pl. 31, fig. 3.
Cryphiops caementarius . – Holthuis 1952a: 137, pls. 33–35. – Holthuis 1952b: 74, fig. 17. – Holthuis 1955: 52, fig. 28. – Bahamonde 1957: 7. – Hartmann 1957: 117. – Hartmann 1958: 15, figs 1–5. – Bahamonde 1962: 7. – Del Solar et al. 1970: 19 (catalog). – Chirichigno Fonseca 1970: 16 (list), fig. 28. – Boschi 1977: 7. – Manning and Hobbs Jr. 1977: 158 (list). – Retamal 1977: 4 (table), 5, fig. 4. – Viacava et al. 1978: 161. – Holthuis 1980: 81 (list). – Méndez 1981: 14 (list), 73 (list), 75 (key), pl. 33 figs 246, 247. – Retamal 1981: 14 (list), fig. 35. – Rodríguez 1981: 47 (list). – Pérez Farfante 1982: 375. – Pretzmann 1983: 316. – Wicksten and Hendrickx 1992: 7 (list). – Holthuis 1993: 106, fig. 93. – Pereira 1997: 21, fig. 18C, 47, table 6 (list). – Bahamonde et al. 1998: 93. – Kameya et al. 1998: 90 (list). – Meléndez and Maldonado 1999: 125, 130. – Jayachandran 2001: 24. – Retamal and Jara 2002: 195, 204 (list). – Zuñiga Romero 2002: 21, 1 fig. – Wicksten and Hendrickx 2003: 60 (list). – Jara et al. 2006: 42, table I, 43, table II, 46, table IV. – Meruane et al. 2006: 285, fig. 1. – Báez and López 2010: 244. – Retamal and Moyano 2010: 307, table 1. – Pileggi and Mantelatto 2010: 197, table 1. – Ríos-Escalante et al. 2013: 850, table 1. – Rossi and Mantelatto 2013: 3, table 1 (list). – Morales and Meruane 2013: 1441, figs 1, 3–5. – Moscoso 2014: 12 (list), 44. – De Grave et al. 2015a: 5, table 1. – Zacarías Ríos and Yépez Pinillos 2015: 398, fig. 1. – Wasiw G., Yépez P. 2015: 166, fig. 2D. – Mantelatto et al. 2020: 915 (key). – Velásquez et al. 2020: 1062.
Cryphiops spinolosomanus . – Maccagno and Cucchiari 1957: 213.
Cryphiops (Cryphiops) caementarius . – Villalobos Hiriart et al. 1989: 162. – De Grave and Fransen 2011: 316 (catalog), fig. 36. – Ashelby et al. 2012: 295, table 1 (list).

###### Material examined.

Chile – **Coquimbo** • 2 males, cl 28.2, cl 36.4 mm; río Limari, Jul. 2006; C. Gaymer leg.; CCDB 1870 • 2 males, cl 30.3, cl 86.5 mm; Limari, río Puente; 19 Oct. 2007; L.G. Pileggi, E.C. Mossolin leg.; CCDB 2146 • 2 males, cl 5.4, cl 5.7 mm, 4 females, cl 5.6 to 10.1 mm, 2 ovigerous females, cl 14.9, cl 16.8 mm, 8 juveniles, cl 3.4 to 4.8 mm; La Serena, Playa el Faro, Avenida de Mar; 18 Oct. 2007; F.L. Mantelatto, L.G. Pileggi, E.C. Mossolin; CCDB 2327.

###### Description.

***Rostrum*.** Straight, short, nearly reaching first article of antennular peduncle; upper margin with 6–8 teeth, regularly spaced, one and/or two behind posterior margin of orbit; lower margin with 0–4 teeth.

***Cephalon*.** Scaphocerite 2 × as long as wide; outer margin convex proximally.

***Carapace*.** Smooth, with strong, acute antennal spine; hepatic spine absent. Lower orbital angle obtuse, moderately pronounced.

***Pereiopods*.**P1 slender, reaching with most of carpus beyond scaphocerite; fingers slightly longer than palm; carpus slightly shorter than chelae; ischium and merus distinctly spinulated; carpus and chelae smooth. P2 strong, with many spines, strong heterochely; largest cheliped reaching with half-length of merus beyond scaphocerite; ischium larger than half-length of merus; merus longer than carpus; carpus short, slightly shorter than half length of palm, with strong basal constriction; propodus 2.1 × as long as dactylus, 3.3 × as long as carpus; palm slightly inflated, more than 2.3 × as long as high; fingers shorter than palm, with numerous small spinules, cutting edges with 4–7 denticles of equal size. P3–P5 smooth, except for sparse setae and spinules along lower margin of propodus; propodus nearly 2 × as long as carpus; propodus slightly shorter than merus; P3 reaching with half-length of dactylus beyond scaphocerite, propodus 2 × as long as dactylus; P4 reaching with tip of dactylus end of scaphocerite, propodus 1.5 × as long as dactylus; P5 reaching with tip of dactylus half-length of scaphocerite, propodus 1.5 × as long as dactylus.

***Pleon*.** Smooth. Somite 5 with posteroventral angle of pleuron acute; somite 6 slightly longer than somite 5. Inter-uropodal sclerite with strong, keel-shaped pre-anal carinae.

***Pleopods*.**PL2 with appendix masculina 2 × as long as appendix interna.

***Uropods*.** Exopodite with mobile spines slightly longer than spiniform projection of outer margin.

***Telson*.** Broad, smooth; 1.5 × as long as abdominal somite 6, bearing 2 pairs of dorsal spinules, first pair located in middle of telson, second pair located ¾ of length of telson. Posterior margin rounded, ending in truncated tip, with several plumose setae and two pairs of posterior spinules, inner pair not reaching end of telson.

###### Size.

See in material examined.

###### Color.

Yellowish green with light brown spots dorsally. P2 with reddish joints and greenish blue color.

###### Type locality.

Chile.

###### Distribution.

Pacific coastal river basins from Perú and Chile (Holthuis 1952a, b; Jara et al. 2006; Morales and Meruane 2013).

###### Life cycle.

Exclusive of coastal waters, dependent of brackish waters to complete its life cycle. The eggs are numerous and small: 0.43–0.62 mm of major diameter (Norambuena 1977; Yávar and Dupré 2007; Bazán et al. 2009). The larval development is long, with many free-swimming larval stages (Morales et al. 2006), following the usual pattern of coastal palaemonid species.

###### Remarks.

For the heterochelia, the robustness and strong shape, as well as the ornamentation of the second pereiopod, *M. caementarius* comb. nov. is comparable with *M. hancocki* Holthuis, 1950, and *M. occidentale* Holthuis, 1950 from the Pacific slope. The species is morphologically similar to *M. heterochirus* (Wiegmann, 1836) from the Atlantic slope, particularly concerning the shape of the rostrum, carapace, and telson.

##### Macrobrachium candango

Taxon classificationAnimaliaDecapodaPalaemonidae

nom. nov. comb. nov.

F55D7A70-4C73-5A8A-A5BF-CA002EE520C9

Cryphiops brasiliensis Gomes Corrêa, 1973: 169, figs 1–26. – Rodríguez 1981: 47 (in list). – Coelho and Ramos-Porto 1985: 407, 409 (table II). – Ramos-Porto and Coelho 1990: 98. – Pereira 1997: 21, fig. 18B, 47, table 6 (in list). – Ramos-Porto and Coelho 1999: 330 (catal.). – Melo 2003: 332, figs 180, 181. – Pileggi and Mantelatto 2010: 197 (table 1). – Mantelatto et al. 2016: 261 (in list). – Mantelatto et al. 2020: 915 (in key), fig. 23.102C.
Cryphiops (Bithynops) brasiliensis . – Villalobos Hiriart et al. 1989: 164, fig. 6b, d. – De Grave and Fransen 2011: 316 (catal.).

###### Material examined.

***Holotype***: Brazil – **Distrito Federal** • male, cl 18.2 mm; Brasília, riacho da Granja do Ipê; 13 Sep. 1966; Emílio Varolli (SUDEPE) leg.; M.M.G. Corrêa det.; MNRJ 903.

###### Additional material examined.

Brazil – **Distrito Federal** • 1 ovigerous female, cl 15.6 mm, [allotype]: Brasília, riacho da Granja do Ipê; 13 Sep. 1966; Emilio Varolli (SUDEPE) leg.; M.M.G. Corrêa det.; MNRJ 6464 • 1 male, cl 17.93 mm, 2 females, cl 15.3, cl 15.3 mm; Brasília, riacho da Granja do Ipê; 23 Feb. 1972; M.M.G. Corrêa leg.; MNRJ 2668 • 1 male, cl 14 mm, 2 females, cl 12.6, cl 13.8 mm; Brasília, córrego Taquara, Reserva Ecológica do IBGE (Instituto Brasileiro de Geografia e Estatística); 05 Aug. 2014, F.L. Mantelatto, L.G. Pileggi, F.L. Carvalho leg.; CCDB 5894 • 2 males, cl 22.9, cl 23.9 mm; Brasília, córrego Onça, upper Paraná River basin; 18 Aug. 1988; E.C. Lopes leg.; CCDB 5895 • 2 males, cl 21.7, cl 24.4 mm; Brasília, córrego Taquara, Onça, upper Paraná River basin; 18 Aug. 1988; E.C. Lopes leg.; CCDB 5896 • 2 females, cl 13.8, cl 14 mm, 5 juveniles, cl 4.4 to 6.2 mm; Brasília, riacho da Granja do Ipê, rio Roncador, Reserva Ecológica do IBGE (Instituto Brasileiro de Geografia e Estatística); 05 Aug. 2014, F.L. Mantelatto, L.G. Pileggi, F.L. Carvalho leg.; CCDB 5897 • 2 males, cl 9.49, cl 10.23 mm, 2 females, cl 11.34, cl 11.70 mm, 4 juveniles, cl 4.55 to 6.30 mm; Brasília, riacho da Granja do Ipê, 22 Feb. 2008, F.L. Mantelatto, E.C. Mossolin leg.; CCDB 2195.

###### Description of the holotype.

***Rostrum*.** Moderately high, nearly straight, distal end slightly directed upwards, reaching end of antennular peduncle, and little before the distal margin of scaphocerite; upper margin convex over orbit, with seven teeth, first and sometimes the second, slightly behind posterior edge of orbit; lower margin with one tooth.

***Cephalon*.** Scaphocerite 2.5 × as long as wide; outer margin slightly concave. Epistome forming two lobes; lobes with laterally arranged carinae.

***Carapace*.** Anterolateral region slightly roughened; hepatic spine absent. Lower orbital angle obtuse, strongly pronounced.

***Pereiopods*.**P1 reaching with almost half length of carpus beyond scaphocerite; fingers as long as palm; carpus 1.5 × as long as chelae, 1.5 × as long as merus; articles with scattered setae, fingers with tufts of setae. P2 similar in shape, different in size; largest one reaching with distal portion of merus beyond scaphocerite; smallest one reaching with distal end of carpus beyond scaphocerite, with fingers as long as palm; all articles with sparse setae and spines. Larger cheliped with ischium nearly as long as merus, with spinulation as in palm; merus as long as carpus, swollen, with spinulation as in palm; carpus slightly shorter than palm, swollen, with strong basal constriction; spinulation as in palm; propodus 2.5 × as long as dactylus, 2 × as long as carpus; palm with upper surface slightly compressed, somewhat swollen, covered with spinules, nearly 3 × as long as high; fingers 2/3 as long as palm, with numerous spinules; cutting edge of dactylus with large tooth in proximal third, slightly lower tooth in between large tooth and proximal part; cutting edge of fixed finger with tooth opposing two teeth of dactylus, with row of three denticles between proximal part and this tooth. P3–P5 smooth, except for sparse setae and spinules along lower margin of propodus; propodus nearly 2 × as long as carpus; propodus slightly shorter than merus; P3 reaching with half-length of dactylus beyond scaphocerite, propodus 2 × as long as dactylus; P4 reaching with tip of dactylus end of scaphocerite, propodus 2.5 × as long as dactylus; P5 reaching with tip of dactylus half-length of scaphocerite, propodus 2.5 × as long as dactylus.

***Pleon*.** Smooth. Somite 5 with posteroventral angle of pleuron rectangular, not spinose; somite 6 1.5 × as long as somite 5. Inter-uropodal sclerite with strong, keel-shaped pre-anal carinae.

***Pleopods*.**PL2 with appendix masculina 2 × as long as appendix interna.

***Uropods*.** Exopodite with mobile spines slightly shorter than spiniform projection of outer margin.

***Telson*.** Broad, smooth, 1.5 × as long as abdominal somite 6, bearing two pairs of dorsal spinules, first pair slightly behind middle portion of telson, second pair located closer to first pair than to posterior margin. Posterior margin distinct, ending in acute point, with several plumose setae and two pairs of posterior spinules, inner pair reaching end of telson.

###### Etymology.

The specific epithet *brasiliensis* was used by Gomes Corrêa (1973) to refer to the type locality of the species, Brasília, the capital of Brazil. To keep that intention, we rename the species using the word *candango*, a demonym referring to those who are native to Brasília.

###### Size.

See in material examined.

###### Color.

From colorless to light brown, with dark brown carapace, mimicking the color of the substrate where they inhabit.

###### Type locality.

Brazil, Distrito Federal, Brasília, Riacho da Granja do Ipê.

###### Distribution.

Endemic of inland waters from Central Brazil (Distrito Federal) (Gomes Corrêa 1973; present paper).

###### Life cycle.

Exclusive of inland waters, therefore independent of brackish waters to complete its life cycle. The fecundity is low, 38–61 eggs, and the eggs are large, their volume ranged from 4.41 to 7.71 mm^3^ (Nogueira et al. under revision). Its larval development is not known but given its fecundity and egg size, it should be abbreviated, following the same pattern of congeners inhabiting continental waters (Magalhães and Walker 1988; Pereira and García 1995).

###### Remarks.

Gomes Corrêa (1973) named *Cryphiops brasiliensis* a species from the vicinities of Brasília, Brazil. This specific epithet, however, was already used by Heller (1868) for a species of *Macrobrachium* described from the state of Mato Grosso, Brazil. With the synonymization of both genera, these specific names become secondary homonyms. We, therefore, propose the name *Macrobrachium candango* nom. nov., comb. nov. as a replacement name for *Cryphiops brasiliensis* Gomes Corrêa, 1973.

We examined specimens from three lots used by Gomes Corrêa (1973) to describe *C. brasiliensis* and deposited at the MNRJ: the holotype (MNRJ 903: male, cl 18.2 mm) and two others labeled as allotype (MNRJ 6464: 1 ovigerous female, cl 15.6 mm) and paratypes (MNRJ 2668: 1 male, cl 17.9 mm, 2 females, cl 15.3 and cl 15.3 mm), although the author did not explicitly designate the latter two as type material. We had this material on loan, which was returned to MNRJ in July 2008. After the fire at the Museu Nacional do Rio de Janeiro in September 2018, the lot MNRJ 2668 is missing, but the other two, including the holotype, preserved in alcohol, are safe and in good condition (I.A. Cardoso, curator of Crustacea, pers. comm. to FLM, Nov 2020). When carrying out aquatic surveys in the region around the type locality, we (FLM, LGP) visited the Reserva Ecológica do IBGE (Brasília, DF) and found a well-preserved collection of specimens (> 260, not listed herein) made during previous aquatic faunistic surveys in the area (Takahashi et al. 2019). The main area of occurrence of this species is in a protected reserve, which may avoid possible impacts. This species was classified in the IUCN’s Data Deficient (DD) category (Mantelatto et al. 2016). However, due to anthropic pressures in the region, future monitoring is necessary to evaluate its conservation conditions.

##### Macrobrachium luscus

Taxon classificationAnimaliaDecapodaPalaemonidae

(Holthuis, 1973) comb. nov.

CD580649-0B79-5C4C-A81D-7FDE955D1687

Bythinops luscus Sbordoni, Argano & Zullini, 1973: 24 (nomen nudum).
Bithynops luscus Holthuis, 1973: 136, figs 1, 2. – Holthuis 1977: 181. – Hobbs, Hobbs and Daniel 1977: 25 (key), 46, fig. 17. – Reddell 1981: 108, fig. 15 (map), 319 (in list), 323 (list). – Villalobos 1982: 217 (in list). – Fitzpatrick 1983: 217. – Holthuis 1986: 606 (list). – Holthuis 1993: 103, fig. 89. – Hobbs III 1993: 20 (list). – Álvarez et al. 1996: 110, chart 12.2 (list). – Pereira 1997: 47, table 6 (list). – Fransen et al. 1997: 15 (catalog). – Jayachandran 2001: 17, fig. 1. – Palacios-Vargas 2006: 7 (list). – Mejía-Ortiz et al. 2013: 32, table 1. – Lamoreux et al. 2015: 306, Appendix 2 (list). – De Grave et al. 2015b: 7, table 3.
Cryphiops (Bithynops) luscus . – Villalobos Hiriart et al. 1989: 163, figs 1, 6a, c, 8a. – Villalobos-Hiriart et al. 1993: 281, table 5 (list). – Hobbs III 1994: 98 (list). – Fransen et al. 2010: 30, Appendix III (list). – De Grave and Fransen 2011: 316 (catalog). – Álvarez et al. 2011: 258, fig. 4a. – Palacios-Vargas and Reddell 2013: 43 (list). – Palacios-Vargas et al. 2014–2015: 22. – Quiroz-Martínez et al. 2014: table S1 (list). – Alvarez and Villalobos 2016: 250, table 8.1 (list).
Cryphiops luscus . – Palacios-Vargas 2006: 7 (list). – Baldari et al. 2010: 48, fig. 1 (map), 52, table 1. – Botello and Alvarez 2013: table 1 (list). – Mantelatto et al. 2020: 915 (key).

###### Material examined.

Mexico – **Chiapas** • 8 males, tl 30.5–49.4 mm, 15 ovigerous females, tl 30.8–46.3 mm; Municipality of La Trinitaria, Rancho de San Rafael del Arco, Gruta del Arco; 07 Apr. 1986; J.L. Villalobos leg.; CNCR 5759.

###### Description.

***Rostrum*.** Short, directed slightly downwards, tip directed slightly upwards, reaching or slightly overreaching joint between second and third article of antennular peduncle, and at level of distal third of scaphocerite; upper margin convex over orbit, with 5–8 teeth regularly spaced, first over or slightly behind posterior edge of orbit; lower margin with none or one tooth.

***Cephalon*.** Scaphocerite 2.5 × as long as wide; outer margin straight.

***Carapace*.** Smooth, with minute punctuations; antennal spine small, slightly overreaching lower portion of orbit; hepatic spine absent. Lower orbital angle obtuse, moderately pronounced.

***Pereiopods*.**P1 slender, reaching with nearly entire chelae beyond scaphocerite; fingers slightly longer than palm; chelae 2/3 length of carpus. P2 moderately robust, with several spines, equal in form and size, reaching with proximal third of carpus beyond scaphocerite; ischium evidently shorter than merus; merus as long as carpus; carpus as long as palm, with basal constriction; propodus 2 × as long as dactylus, 2 × as long as carpus; palm inflated, nearly 3 × as long as high; fingers as long as palm, with numerous small spinules; cutting edge with two denticles of same size in both teeth. P3–P5 with all joints covered with row of small spinules on the lower margin; P3 reaching with entire dactylus beyond scaphocerite, propodus 2 × as long as dactylus, propodus slightly longer than merus; P4 reaching with tip of dactylus end of scaphocerite, propodus 3 × as long as dactylus, propodus slightly longer than merus; P5 reaching with tip of dactylus half-length of scaphocerite, propodus 3 × as long as dactylus, propodus slightly longer than merus.

***Pleon*.** Smooth. Somite 5 with posteroventral angle of pleuron acute; somite 6 nearly 2 × as long as somite 5. Inter-uropodal sclerite without, keel-shaped pre-anal carinae.

***Pleopods*.**PL2 with appendix masculina 2 × as long as appendix interna.

***Uropods*.** Exopodite with mobile spines as long as spiniform projection of outer margin.

***Telson*.** Broad, smooth, slightly longer than abdominal somite 6, bearing two pairs of dorsal spinules closer to posterior margin of telson. Posterior margin ending in moderately acute triangular point, with several plumose setae and two pairs of posterior spinules, inner pair overreaching end of telson.

###### Size.

See in material examined.

###### Color.

Whitish to transparent.

###### Type locality.

México, Chiapas, Municipality of La Trinitaria, Gruta del Arco, El Rancho de San Rafael Del Arco, Lagunas de Montebello, altitude 1,470 m. Recent visits to the type locality showed an increasing contamination in the lakes that supply water to the underground stream of the Gruta del Arco, and the collections of specimens were not successful, at least in the closest access to the water pools. Possibly, *M. luscus* comb. nov. is seriously threatened.

###### Distribution.

Only known from the type locality (Holthuis 1973; present paper).

###### Life cycle.

This is a cave species exclusive of inland waters, therefore independent of brackish to complete its life cycle. The eggs are few and large: 1.8–2.4 mm (Villalobos Hiriart et al. 1989). The duration of the embryonic development is probably long and with few larval stages following the pattern of other inland species.

###### Remarks.

This species is similar to *Macrobrachium valdonii* nom. nov., comb. nov., which is the other hypogean species with abbreviated development and without hepatic spine. The most remarkable differences between them concerns the length of the rostrum, and the proportion of the articles of second pereiopod (Table 2). In *M. luscus* comb. nov., the rostrum is short, reaching or slightly overreaching joint between second and third article of antennular peduncle, and at level of distal third of scaphocerite. The ischium of the second pereiopod is evidently shorter than the merus, and the dactyl is little longer or as long as palm. In *M. valdonii* nom. nov., comb. nov., the rostrum is longer, reaching the third article of antennular peduncle and the distal border of scaphocerite; the ischium of the second pereiopod is slightly shorter than the merus, and the dactyl is slightly shorter than the palm.

##### Macrobrachium perspicax

Taxon classificationAnimaliaDecapodaPalaemonidae

(Holthuis, 1977) comb. nov.

F151D75D-37E8-5657-8F7D-52F400C2FFD9

Bithynops perspicax Holthuis, 1977: 182, figs 3, 4. – Reddell 1981: 108, fig. 15 (map). – Villalobos 1982: 217 (list). – Holthuis 1986: 606 (list). – Álvarez et al. 1996: 110, chart 12.2 (list). – Pereira 1997: 47, table 6 (list). – Fransen et al. 1997: 16 (catalog). – Jayachandran 2001: 17. – Mejía-Ortiz et al. 2013: 32, table 1 (list).
Bithinops perspicax . – Sbordoni et al. 1977: 52, pl. 3 [error].
Cryphiops (Bithynops) perspicax . – Villalobos Hiriart et al. 1989: 165, figs 1, 7a, c, 8b. – Villalobos-Hiriart et al. 1993: 281, table 5 (list). – Hobbs III 1994: 98 (list). – Fransen et al. 2010: 30, Appendix III (list). – De Grave and Fransen 2011: 316 (catalog). – Álvarez et al. 2011: 258, fig. 2c. – Palacios-Vargas and Reddell 2013: 43 (list). – Palacios-Vargas et al. 2014–2015: 22. – Quiroz-Martínez et al. 2014: table S1 (list). – Alvarez and Villalobos 2016: 250, table 8.1 (list).
Cryphiops perspicax . – Palacios-Vargas 2006: 7 (list). – Baldari et al. 2010: 48, fig. 1 (map), 52, table 1. – Mantelatto et al. 2020: 916 (key).

###### Material examined.

Mexico – **Chiapas** • 16 males, tl 31.1–43.3 mm, 16 ovigerous females, tl 21.6–35.5 mm; Municipality of La Trinitaria, Ruinas de Chincultik, Cenote La Cueva; 07 Apr. 1986; J.L. Villalobos-Hiriart, J.C. Nates-Rodríguez, A. Cantú-Díaz Barriga leg; CNCR 7898.

###### Description.

***Rostrum*.** Short, directed downwards, reaching joint between second and third articles of antennular peduncle; upper margin with 5–8 teeth regularly spaced, first one at level or slightly behind posterior edge of orbit; lower margin with 1–3 teeth.

***Cephalon*.** Scaphocerite 2.6 × as long as wide, outer margin straight.

***Carapace***: Smooth, with minute punctuations; antennal spine small, slightly overreaching lower portion of orbit; hepatic spine absent. Lower orbital angle subacute, moderately pronounced.

***Pereiopods*.**P1 slender, reaching with entire chelae or small part of carpus beyond scaphocerite; fingers slightly longer than palm; chelae 2/3 length of carpus. P2 moderately robust, with spines, equal in form and size, reaching with proximal third of carpus beyond scaphocerite; ischium evidently shorter than merus; merus as long as carpus; carpus as long as palm, with basal constriction; propodus 2.2 × as long as dactylus, 2 × as long as carpus; palm inflated, nearly 3 × as long as high; fingers slightly shorter (0.8) than palm, with numerous small spinules, not gaping, tips crossing, cutting edges with two similar denticles closer to proximal portion. P3–P5 with all joints covered with row of small spinules on lower margin; P3 reaching with entire dactylus beyond scaphocerite, propodus 2 × as long as dactylus, propodus nearly 2 × as long as carpus, propodus slightly longer than merus; P4 reaching with tip of dactylus end of scaphocerite, propodus 3 × as long as dactylus, propodus nearly 2 × as long as carpus, propodus slightly longer than merus; P5 reaching with tip of dactylus half-length of scaphocerite, propodus 3 × as long as dactylus, propodus nearly 2 × as long as carpus, propodus slightly longer than merus.

***Pleon*.** Smooth, somite 5 with posteroventral angle of pleuron acute; somite 6 nearly 2 × as long as somite 5. Inter-uropodal sclerite without keel-shaped pre-anal carinae.

***Pleopods*.**PL2 with appendix masculina nearly 2 × as long as appendix interna.

***Uropods*.** Exopodite with mobile spines as long as spiniform projection of outer margin.

***Telson*.** Broad, smooth, slightly longer than abdominal somite 6, bearing two pairs of dorsal spinules close to posterior margin of telson. Posterior margin ending in moderately acute triangular point, with several plumose setae and two pairs of posterior spinules, inner pair overreaching end of telson.

###### Size.

See in material examined.

###### Color.

Body translucid with orange punctuations.

###### Type locality.

México, Chiapas, Municipality of La Trinitaria, Cenote La Cueva, Ruinas de Chincultik, altitude 1,480 m.

###### Distribution.

Only known from the type locality (Holthuis 1977; present paper).

###### Life cycle.

Exclusive of inland waters, therefore independent of brackish waters to complete its life cycle. The eggs are few and large: 1.9–2.5 mm (Villalobos Hiriart et al. 1989). Its larval development is not known but given the characteristics of the eggs, it should be abbreviated, following the same pattern of congeners inhabiting continental waters (Magalhães and Walker 1988; Pereira and García 1995).

###### Remarks.

Among the epigean forms of this group of species with abbreviated development and without hepatic spine, *M. perspicax* comb. nov. can be distinguished from *M. candango* nom. nov., comb. nov. and *M. alevillalobosi* nom. nov., comb. nov. by the total length of the body, and by the similar form and size of the second pereiopod and the proportion of its articles (Table 2). Specimens of *M. perspicax* comb. nov. are generally smaller (31.1–43.3 mm) than those of the other two species; the second pereiopods are shorter, do not present heterochely like *M. candango* nom. nov., comb. nov. and the chelae are slender, the palm is 3 × as long as high, and the dactylus is slightly shorter.

##### Macrobrachium valdonii

Taxon classificationAnimaliaDecapodaPalaemonidae

nom. nov. comb. nov.

29ABADC2-37C6-58B2-83FC-64B499510C4D

Cryphiops sbordoni Baldari, Mejía-Ortíz & López-Mejía, 2010: 48, figs 2–4. – Mantelatto et al. 2020: 915 (key).
Cryphiops (Bithynops) sbordonii . – De Grave and Fransen 2011: 316 (catalog). – Palacios-Vargas and Reddell 2013: 43 (in list). – Palacios-Vargas et al. 2014–2015: 22. – Quiroz-Martínez et al. 2014: table S1 (list). – Alvarez and Villalobos 2016: 250, table 8.1 (list).

###### Material examined.

***Holotype***: Mexico – **Chiapas** • male, cl 25 mm; Las Margaritas, Cueva Chamburro; 01 Mar. 2001; V. Sbordoni leg.; CNCR 25106. ***Paratypes***: 1 ovigerous female, cl 22.5 mm, allotype; same data as for holotype; CNCR 25107 • 1 female, cl 12.3 mm; same data as for holotype; CNCR 25108.

###### Description.

***Rostrum*.** Short, straight, tip not reaching distal border of scaphocerite, almost reaching third article of antennular peduncle; upper margin bearing eight teeth, lower margin smooth.

***Cephalon*.** Eyes reduced, globular cornea with facets, pigmented area reduced to a black point. Scaphocerite 2.4 × as long as wide.

***Carapace*.** Smooth, maximum length 25 mm, with only antennal spine; branchiostegal groove shallow; hepatic spine absent.

***Pereiopods*.**P1: slender, smooth, with few tufts of setae on both fingers; palm surpassing distal margin of scaphocerite; palm slightly compressed, as long as dactylus; carpus 1.75 × as long as palm, 1.12 × as long as merus. P2: subequal in size, subequal in size, reaching with half of carpus beyond scaphocerite, without spines; ischium 0.9 × merus; carpus 0.8 × as long as merus, 0.85 × as long as palm; propodus 1.5 × as long as dactylus, 2.5 × as long as carpus; palm semi-cylindrical, 3.3 × as long as high, with dispersed tufts of setae, 0.8 × as long as dactylus; fingers elongated, not gaping, cutting edges covered with tufts of setae, dactylus without teeth. P3: propodus, dactylus with several short setae, row of seven spines on ventral margin, propodus 3 × as long as dactylus, 2.05 × as long as carpus. P4: sparsely pilose, propodus 3.4 × as long as dactylus, 1.8 × as long as carpus, propodus with row of nine movable spines on ventral margin, propodus-dactylus articulation with pair of setae. P5: longest, propodus, carpus pilose, with longitudinal row of 12 movable spines, distal four close together, propodus-dactylus articulation with one spine; propodus 4 × as long as dactylus, 2.1 × as long as carpus.

***Pleon*.** Smooth; somites 1–3 with pleura broadly rounded; somites 4 and 5 with posteroventral margin of pleura rounded; pleura of all somites bearing setae on ventral border; somite 6 nearly 1.5 × as long as somite 5. Inter-uropodal sclerite without keel-shaped pre-anal carinae.

***Telson*.** Nearly 1.5 × longer than abdominal somite 6, shorter than uropodal rami, bearing two pairs of dorsal spines, first pair on distal fifth, second pair on middle section, with a single spine in the middle on left side; posterior margin broadly triangular bearing two pairs of lateral spines, inner pair 5 × longer than outer one, with plumose setae between inner spines, center ending in acute tip.

###### Etymology.

Baldari et al. (2010) named this species in honor of Prof. Valerio Sbordoni, a studious of the cave fauna of Chiapas, Mexico, and collector of the specimens. We maintained this homage by forming the specific epithet with parts of his first and last name.

###### Size.

See in material examined.

###### Color.

Live specimens are white, without pigment in/on the body.

###### Type locality.

Mexico Chiapas, Las Margaritas, Cueva Chamburro.

###### Distribution.

Only known from the type locality (Baldari et al. 2010).

###### Life cycle.

Stygobitic species exclusive of inland waters, therefore independent of brackish waters to complete its life cycle. Female allotype with eggs (not measured).

###### Remarks.

Mejía-Ortíz et al. (2008) described *Macrobrachium sbordonii* from Mexico, naming it after Valerio Sbordoni. Shortly thereafter, Baldari et al. (2010) pay homage to the very same person again by describing a new species of *Cryphiops* also from Mexico. Since the synonymization of both genera makes the names secondary homonyms, *Macrobrachium valdonii* nom. nov., comb. nov. is proposed as a replacement name for *Cryphiops sbordonii* Baldari, Mejía-Ortiz & López-Mejía, 2010.

Similar to *M. luscus* comb. nov. (see remarks of that species and Table 2).

## Discussion

### Taxonomic issues

The phylogenetic analysis presented here, including freshwater prawns of the genus *Cryphiops* and species of *Macrobrachium* from four different geographic regions revealed that they form an unnatural group inside the Palaemonidae. All the species of *Cryphiops*, however, were considered valid taxonomic entities and all of them were recovered in the proper group of *Macrobrachium* species in terms of distribution and type of larval development.

*Macrobrachium caementarius* comb. nov. was consistently recovered associated to species with an estuarine affinity, supporting the taxonomic similarity showed in the phylogenetic analysis. The endemic species from Mexico, *Macrobrachium luscus* comb. nov., *M. perspicax* comb. nov., *M. valdonii* nom. nov., comb. nov., and *M. alevillalobosi* nom. nov., comb. nov., appear to have a joint position, always close to the species of *Macrobrachium* from Mexican inland waters (Fig. 1), which confirms the phylogenetic relationships among the four species. Similarly, the endemic species from central Brazil, *M. candango* nom. nov., comb. nov. is related to species of *Macrobrachium* also endemic to Brazil, in particular *M. iheringi* (Fig. 1), with a high degree of morphological similarity between these species.

The results of the taxonomic analysis of the species of *Cryphiops* corroborated the findings reported by Holthuis (1950, 1952a), who listed a series of morphological and biological reasons to explain why the taxonomy of the genera within the family Palaemonidae is considered of difficult resolution and deserved more refined studies. Therefore, it is not surprising that the current systematics of the group used so far exhibited several inconsistencies at both the generic and specific levels, such as those already reported for other species when molecular analysis were contrasted with morphologically based classifications (Murphy and Austin 2002, 2003).

The morphological character used to define *Cryphiops* is clear and easily discernible: “This genus differs from *Macrobrachium*, with which it often is united, mainly by the absence of the hepatic spine on the carapace” (see Holthuis 1952a: 136). That is, the only synapomorphy separating the two genera is the absence of the hepatic spine in *Cryphiops*. In accordance with Short (2004), the presence or absence of a hepatic spine is a doubtful character in Palaemonidae because it sometimes can be absent from one or both sides in specimens of *Macrobrachium*. Therefore, this single character used to separate *Cryphiops* is subjective, and its usefulness should be reconsidered. Clearly, the absence of the hepatic spine refers to a case of homoplasy, in which the independently acquired apomorphies do not represent phylogenetic proximity. In this case, two hypotheses can be considered: 1) parallelism, losing the hepatic spine independently in the two lineages from a plesiomorphic with-hepatic-spine state, or 2) reversal, when the apomorphic state (absence of hepatic spine) becomes similar to the previous plesiomorphic state (absence of hepatic spine) present in the ancestor of the group. From a parsimony point of view, however, we believe that the first hypothesis seems more plausible, i.e., an independent loss of the hepatic spine that was propagated from generation to generation in different populations.

### Nomenclatural issues

The obtained concatenated topology (Fig. 1) shows that there is high genetic similarity among the species of *Macrobrachium* and *Cryphiops*, coinciding with several previous studies that suggested that the latter should be part of *Macrobrachium* (Pereira 1997; Porter et al. 2005; Page et al. 2008; Pileggi and Mantelatto 2010). Following these studies, the robust results obtained here, considering all species of *Cryphiops* and almost all of the Neotropical species of *Macrobrachium*, corroborate the paraphyletic nature of these groups and indicate that the current classification should be amended accordingly. In this way, as De Grave and Ashelby (2013: 341) pointed out, such amendment will induce a nomenclatural issue regarding the priority of the names *Cryphiops* / *Macrobrachium*, a situation that demands extra caution and that will require an evaluation by the International Commission on Zoological Nomenclature (ICZN). The name *Cryphiops* Dana, 1852 precedes *Macrobrachium* Spence Bate, 1868 and, if the Principle of Priority is strictly followed, the former should have priority over the latter (ICZN 1999, Art. 23). However, *Macrobrachium* is a much more speciose genus with many species of economic interest and importance, and extensively cited in the scientific literature. Therefore, a change in the generic name that at present is very well known would certainly cause taxonomic confusion and nomenclatural instability. The provisions of the Article 23.9.1 of the Code for a Reversal of Precedence cannot be applied in this case because the older synonym (*Cryphiops*) was used as a valid name after 1899 (see synonymic list under *Macrobrachium*). We, nevertheless, invoke the provision of Article 23.9.3 to propose herein that the younger synonym (*Macrobrachium*) keeps the priority over the older one. An application to the International Commission on Zoological Nomenclature to suppress the priority of *Cryphiops* and rule this proposal of Reversal of Precedence is being concurrently prepared. We also suggest that the prevailing use of the name *Macrobrachium* is maintained while the matter is under consideration by the Commission (ICZN 1999, Art. 82). Meanwhile, those who believe the taxa to be distinct could still use both names (L.B. Holthuis, pers. comm. to FLM on 27 Nov 2007). The arguments to support this proposal are detailed below.

In an essay on Chile’s natural history, Molina (1782) introduced “*Cancer caementarius*” to name a freshwater shrimp abundant in the rivers of that country. This species was later treated under different names or combinations [for instance: *Astacus caementarius* by Molina (1810), *Palaemon caementarius* by Poeppig (1835), *Palaemon gaudichaudii* by H. Milne Edwards (1837, in H. Milne Edwards 1834–1840), *Cryphiops spinuloso-manus* by Dana (1852), *Bithynis longimana* by Philippi (1860), *Macrobrachium africanum* by Spence Bate (1868), and *Bithynis caementarius* by Rathbun (1910); see Holthuis (1952a, b) for a complete synonymy] until Holthuis (1950, 1952b) noted that the specimen described by Dana (1852) was actually a mutilated specimen of *Bithynis caementarius* (Molina, 1782) and pointed out that *Cryphiops* Dana, 1852 had priority over the name *Bithynis* Philippi, 1860. Therefore, Holthuis (1952a) established the type species of the genus as being *Cryphiops spinulosomanus* Dana, 1852 (= *Cryphiops caementarius* (Molina, 1782)).

The genus remained monotypic for more than 120 years until Gomes Corrêa (1973) described *Cryphiops brasiliensis*, endemic to central Brazil. In that same year, Holthuis (1973) erected *Bithynops* to include a new cave species from Mexico, *Bs. luscus*. Soon after, Holthuis (1977) included another new species from Mexico in this genus: *Bithynops perspicax*. Subsequently, in a review of the genera *Cryphiops* and *Bithynops*, Villalobos Hiriart et al. (1989) proposed the synonymization of both taxa based on the fragility of the characters used to separate them (e.g., eyes with reduced cornea in *Bithynops*), but kept both taxa with subgeneric status. They retained *C. caementarius* under *Cryphiops* s. s., moved *C. brasiliensis*, *Bs. luscus*, and *Bs. perspicax* into Cryphiops (Bythynops), in addition to describing a new species, Cryphiops (Bithynops) villalobosi Villalobos Hiriart, Nates Rodriguez & Diaz Cantú, 1989. Later, Baldari et al. (2010) described a new cave species from Chiapas, Mexico, named *Cryphiops sbordonii* Baldari, Mejía-Ortiz & López-Mejía, 2010. It is noteworthy that Holthuis (1993), in his robust review of the caridean genera, did not follow this subgeneric arrangement, which is widely accepted (De Grave and Fransen 2011; WoRMS 2021).

The genus *Macrobrachium* was erected by Spence Bate (1868) to accommodate four species with males presenting “immensely developed” second pair of pereiopods without, however, designating a type species. This was subsequently done by Fowler (1912), who chose an American species, *Macrobrachium americanum* Spence Bate, 1868, as the type species.

Holthuis and Ng (2010) gave a historical overview of the nomenclatural situation of the name *Macrobrachium*, in particular regarding the confusing usage of the names *Macrobrachium* and *Palaemon* Weber, 1795, which led the matter to be ruled by the International Commission of Zoological Nomenclature in the Opinion 564 (ICZN 1959). Due to the very conservative nature of the morphological traits used to differentiate this group of palaemonid shrimps both to generic and specific ranks, the taxonomic status of *Macrobrachium* has undergone several changes, especially until the first half of the 20^th^ century. Spence Bate (1868) confessed his hesitation in creating the new genus, since he did not perceive any structural differentiation separating the new species of *Macrobrachium* from those of *Palaemon* but considered that the extremely long P2 would be a strong evidence that both taxa formed a natural classification. Shortly thereafter, the author did not follow his own system and treated *Macrobrachium* as a junior synonym of *Bithynis* (see Spence Bate 1888: 788). Ortmann (1891), based on characters of the P2 (shape of the palm and length ratio between carpus and merus), split up *Palaemon* into four subgenera: *Eupalaemon* Ortmann, 1891; *Brachycarpus* Spence Bate, 1888; *Parapalaemon* Ortmann, 1891; and *Macrobrachium*. His system was followed by Coutière (1901), but not by Stebbing (1908), who, in view of the inconsistency of such arrangement, argued that the retention of the name *Macrobrachium* was not justified and replaced it with *Macroterocheir*, a genus defined by one of the chelipeds of the second pair being exceedingly longer than the other. Henderson and Matthai (1910) found the subgeneric arrangement of doubtful utility, since those characters were age dependent, and kept all species under the genus *Palaemon*. Holthuis (1950, 1952a) presented a comprehensive discussion on the difficulties of studying the taxonomy of this group regarding the few useful differential characters and their large variability individually, ontogenetically or between the sexes. Holthuis (1950: 104) also considered the subgeneric division unfeasible, as it could lead to confusion, and treated *Macrobrachium* as a unity.

Since Holthuis’ revision (1952a) of the American Palaemoninae and, particularly, after the Opinion 564 (ICZN, 1959), the taxonomic and nomenclatural status of the genus has remained stable. As a pantropical and subtropical genus occurring in a wide variety of habitats, the number of species from around the world added or described in it grew so rapidly that 41 years after his revision, Holthuis (1993) himself remarked that “it is now a quite respectable generic name”. Today, the genus is one of the most speciose of the infraorder Caridea, with 243 valid species until 1 June 2011 (De Grave and Fransen 2011) and 259 until 5 Jan 2021 (WoRMS 2021), with this number varying either due to the frequent addition of new species (e.g., Mejía-Ortíz and López-Mejía 2011; Pillai and Unnikrishnan 2012, 2013; Pillai et al. 2014, 2015; Fujita et al. 2015; Cai and Vidthayanon 2016; Lan et al. 2017; Saengphan et al. 2018, 2019; Xuan 2019; Zheng et al. 2019; Rossi et al. 2020; Siriwut et al. 2020; Zhu et al. 2020; Myo et al. 2021) or due to synonymization or revalidation of species (e.g., Pileggi and Mantelatto 2012; Castelin et al. 2017).

The high diversity and worldwide tropical-subtropical distribution of *Macrobrachium*, combined with the scarcity of morphologic characters for accurate generic and specific delimitation, has long been intriguing taxonomists regarding its systematics, phylogenetic affinities, and biogeographic patterns. Several studies have been published on these topics using both morphological and molecular data, and, more recently, applying integrative approaches (Pereira 1997; Bowles et al. 2000; Murphy and Austin 2003, 2005; Short 2004; Hernández et al. 2007; Liu et al. 2007; Valencia and Campos 2007; Wowor and Ng 2007; Parhi et al. 2008; Wowor et al. 2009; Pileggi and Mantelatto 2010; Acuña Gómez et al. 2013; Rossi and Mantelatto 2013; Pileggi et al. 2014; Jose et al. 2016; Jose and Harikrishnan 2019; Mokambu et al. 2019; Molina et al. 2020). Among other factors, the high number of species has been hampering a comprehensive study on the phylogeny of the genus, but several articles were published on this at a regional level, either based on American (e.g., Pileggi and Mantelatto 2010; Acuña Gómez et al. 2013; Rossi and Mantelatto 2013; Pileggi et al. 2014), African (e.g., Mokambu et al. 2019), or Indo-West Pacific species (e.g., Murphy and Austin 2005; Liu et al. 2007; Parhi et al. 2008; Chen et al. 2009; Wowor et al. 2009; Jose and Harikrishnan 2019; Siriwut et al. 2020). As one of the most conspicuous constituents of the aquatic fauna in estuarine and continental aquatic environments, a multitude of studies on the biology, ecology, reproduction, development, and physiology of many of its species have already been published. Jayachandran (2001) and Anger (2013) made a comprehensive review on the biology, ecology, and biogeography of *Macrobrachium* (see also the references therein).

The large size, high fecundity, and abundance of some species of the genus have made them an economically valuable fisheries and aquaculture resource and, consequently, numerous scientific and technical publications on different aspects related to their culture and fisheries have been made around the world (see New and Valenti 2000; Jayachandran 2001; New et al. 2008, 2010). *Macrobrachium rosenbergii* (De Man, 1879) and *Macrobrachium nipponense* (De Haan, 1849 [in De Haan 1833–1850]) are the most commercially important species, but other species of *Macrobrachium* have also been used for aquaculture or studied as potentially cultivable species (New and Valenti 2000; Jayachandran 2001; New et al. 2008, 2010; Hongtuo et al. 2012; New and Mohanakumaran Nair 2012; FAO 2020).

Holthuis and Ng (2010), considering the circumtropical, disjunct geographic distribution of this highly diverse group, raised doubts as to whether the genus would form a monophyletic clade. To this regard, we included eight Asian and two African species of *Macrobrachium* (Table 1); however, they were recovered either nested within American species or well within what is considered the genus *Macrobrachium* (Fig. 1). Although our study is limited to the available sequences and species that we were able to analyze and sequence, it contributes to the assumption that the genus is monophyletic and is supported by a multigene analysis. Other studies using molecular approaches, but also including a limited number of representatives either with preponderance of Indo-Pacific species (Murphy and Austin 2005; Liu et al. 2007; Parhi et al. 2008; Wowor et al. 2009; Jose and Harikrishnan 2019) or focused on American species (Pileggi and Mantelatto 2010; Acuña Gómez et al. 2013; Rossi and Mantelatto 2013; Pileggi et al. 2014; García-Velazco et al. 2017, 2018), have also pointed to a monophyletic status of the genus. Anger (2013) assumed that all *Macrobrachium* species originated from the same ancestor in proposing a robust scenario for explaining the origin, evolutionary history, and modern biogeography of the genus. Assuming that it is indeed monophyletic and considering that the type species of *Macrobrachium* is an American species, then our proposal of Reversal of Precedence of *Macrobrachium* over *Cryphiops*, if so ruled by the ICZN, should not affect the status and situation of the African and Indo-West Pacific species of *Macrobrachium*. On the other hand, if future, more comprehensive studies including a large number of worldwide representatives of the genus will eventually not corroborate its monophyly, then the taxonomic and nomenclatural situation of the non-American species might become somewhat complicated. Among the other generic names available, *Eupalaemon* Ortmann, 1891 cannot be used because its type species, designated by Holthuis (1955), is *Macrobrachium acanthurus* (Wiegmann, 1836), a well-established American species. If the African and Asian species constitute a separate clade, then *Parapaleomon* Ortmann, 1891 would be the name to be used, as Holthuis (1955) established its type species as being *Macrobrachium dolichodactylus* Hilgendorf, 1879, a species from the eastern coast of Africa (Mozambique). If, however, the results of such a study pose more atomized groups, the introduction of new generic names for those clades might be necessary, since the type species of *Macroterocheir* Stebbing, 1908, the only other name available for this group, is *Macrobrachium lepidactylus* Hilgendorf, 1879 (designated by Holthuis 1955), also from Mozambique.

## Conclusions

Our phylogenetic analysis of all species of *Cryphiops*, including species of *Macrobrachium* from America, Africa, and the Indo-Pacific, using morphological and multigene approaches in combination with a taxonomic revision, revealed that the morphological character used to separate the genus *Cryphiops* is subjective and homoplasic, and that all *Cryphiops* species are nested within *Macrobrachium*. Such results corroborate the assumption about the monophyly of the genus *Macrobrachium*, which implies that *Cryphiops* Dana, 1852 and *Macrobrachium* Spence Bate, 1868 are subjective synonyms and, as a consequence, three specific secondary homonyms are established: *M. brasiliense* (Heller, 1862) × *C. brasiliensis* Gomes Corrêa, 1973; *M. villalobosi* Hobbs Jr, 1973 × C. (Bithynops) villalobosi Villalobos Hiriart, Nates Rodríguez & Cantú Díaz Barriga, 1989; and *M. sbordonii* Mejía-Ortíz, Baldari & López-Mejía, 2008 × *C. sbordonii* Baldari, Mejía-Ortiz & López-Mejía, 2010. We therefore present a systematic rearrangement in which all species of *Cryphiops* are included in *Macrobrachium* and introduce replacement names for the three resulting specific secondary homonyms.

The available genetic data argues for the synonymy of *Macrobrachium* Spence Bate, 1868 under *Cryphiops* Dana, 1852. Considering the large number of species under both names and the fact that they have a pan-tropical distribution, it is likely this taxonomy may be challenged by new genetic techniques and finer morphological analyses. To change the generic names at this stage would be very disruptive, resulting in nomenclatural instability and causing confusion for other researchers, especially since there are several economically important species (notably *Macrobrachium rosenbergii*). Moreover, many species are also important in conservation efforts and used for a wide variety of biological studies in many parts of the world. Therefore, until a larger data set can be assembled, we recommend maintaining the status quo with regards to the generic names, i.e., use *Macrobrachium* sensu lato and restrict the use of *Cryphiops* for *C. caementarius* (Molina, 1782) and its immediately allied species. Under the current code (ICZN 1999: Arts. 23.9.3, 81.2.2), the senior synonym (*Cryphiops*) should be partially suppressed in favor of maintaining the prevailing use of the junior synonym (*Macrobrachium*) under the provision of the Article 82 of the Code (ICZN 1999). In this sense, an application is concurrently being prepared to the ICZN for using their Plenary Powers to partially suppress the priority of the name *Cryphiops* over the name *Macrobrachium* and rule a case of Reversal of Precedence regarding these names.

## Supplementary Material

XML Treatment for Macrobrachium

XML Treatment for Macrobrachium alevillalobosi

XML Treatment for Macrobrachium caementarius

XML Treatment for Macrobrachium candango

XML Treatment for Macrobrachium luscus

XML Treatment for Macrobrachium perspicax

XML Treatment for Macrobrachium valdonii

## Supplementary materials

Supplementary material 1 Table S1 Data type Pairwise genetic distance matrix (excel table) Explanation note Pairwise genetic distance matrix of 16S (below diagonal) and COI (above diagonal) sequences among species previously included in the genus "*Cryphiops*" Dana, 1852 and "selected species" of *Macrobrachium* Spence Bate, 1868 from America, Africa, and the Indo-Pacific. https://binary.pensoft.net/file/561677This dataset is made available under the Open Database License (http://opendatacommons.org/licenses/odbl/1.0/). The Open Database License (ODbL) is a license agreement intended to allow users to freely share, modify, and use this Dataset while maintaining this same freedom for others, provided that the original source and author(s) are credited.Fernando L. Mantelatto, Leonardo G. Pileggi, João A. F. Pantaleão, Célio Magalhães, José Luis Villalobos, Fernando Álvarez
